# Emergence and dynamics of influenza super-strains

**DOI:** 10.1186/1471-2458-11-S1-S6

**Published:** 2011-02-25

**Authors:** Brian J Coburn, Chris Cosner, Shigui Ruan

**Affiliations:** 1 Center for Biomedical Modeling, Semel Institute of Neuroscience & Human Behavior, David Geffen School of Medicine, University of California, Los Angeles, 10940 Wilshire Blvd, Suite 1450, Los Angeles, CA 90024, USA; 2 Department of Mathematics, University of Miami, Coral Gables, FL 33124-4250, USA

## Abstract

**Background:**

Influenza super-strains can emerge through recombination of strains from birds, pigs, and humans. However, once a new recombinant strain emerges, it is not clear whether the strain is capable of sustaining an outbreak. In certain cases, such strains have caused major influenza pandemics.

**Methods:**

Here we develop a multi-host (i.e., birds, pigs, and humans) and multi-strain model of influenza to analyze the outcome of emergent strains. In the model, pigs act as “mixing vessels” for avian and human strains and can produce super-strains from genetic recombination.

**Results:**

We find that epidemiological outcomes are predicted by three factors: (i) contact between pigs and humans, (ii) transmissibility of the super-strain in humans, and (iii) transmissibility from pigs to humans. Specifically, outbreaks will reoccur when the super-strain infections are less frequent between humans (e.g., R*_0_*=1.4) but frequent from pigs to humans, and a large-scale outbreak followed by successive dampening outbreaks will occur when super-strain infections are frequent between humans (e.g., R*_0_*=2.3). The average time between the initial outbreak and the first resurgence varies from 41 to 82 years. We determine the largest outbreak will occur when 2.3 <*R_0_* < 3.8 and the highest cumulative infections occur when 0 <*R_0_* < 3.0 and is dependent on the frequency of pig-to-human infections for lower R_0_ values (0 <*R_0_* < 1.9).

**Conclusions:**

Our results provide insights on the effect of species interactions on the dynamics of influenza super-strains. Counter intuitively, epidemics may occur in humans even if the transmissibility of a super-strain is low. Surprisingly, our modeling shows strains that have generated past epidemics (e.g., H1N1) could resurge decades after they have apparently disappeared.

## Background

Until very recently there has been limited attention focused on the role of pigs in the spread of influenza and even less on the possibility of recombination of avian and human viruses in pigs [1-4]. The emergence of the novel strain of influenza A (H1N1) in Mexico [5,6] in April 2009 has shown birds, pigs and humans can all play a role in the formation of new strains and can be transmitted from pigs to humans [7]. Prior to the outbreak, much of the surveillance against pandemic influenza has been focused on avian strains (e.g., H5N1).

Influenza pandemics occur when a transmissible strain emerges into the human population in which humans have little or no immunity. One way this can occur is when a strain emerges in humans from a reservoir host species (e.g., birds or pigs). In fact, the three previous influenza pandemics (1918 H1N1, 1957 H2N2, and 1968 H3N2) emerged to humans from a non-human reservoir and were subtypes that originated in avian hosts [7]. In fact, strong evidence suggests all influenza subtypes trace back to avian origin, implying the avian virus emerged and established itself in mammals from birds. Avian strains such as the H5N1 have proven to be the most threatening particularly to humans upon infection, killing approximately 50 to 70 percent of those infected [8]. An H5N1 pandemic appears unlikely in that the strain has been shown to transmit poorly between humans. Furthermore, a strain with such high virulence would also need to be highly transmissible in order for a pandemic to occur.

Genetic changes in influenza provide new opportunities for pathogen emergence in humans, particularly through recombination. When coinfection occurs, two strains may interact and recombine to form a new reassortant strain. Intermediate hosts, such as pigs and domestic poultry, play an important role in influenza infection between birds and human. Pigs are also capable of infection by human influenza viruses [9]. It has been reported that pigs are capable of infecting humans [4,10,11] and are likely candidates as intermediate hosts for coinfection of interspecies strains [12].

Several empirical studies indicate that not only avian and human influenza strains are likely to establish infection in pigs but also that pigs are capable of passing newly reassortant strains to humans. Strikingly, half of Java’s pigs in 2005 were reported to be infected with avian influenza H5N1 [13]. In 1999, a strain of avian origin (H4N6) was isolated from pigs in Canadian swine farms [14]. Furthermore, the previously known H1N1 swine virus emerging in Europe in 1979 was antigenically similar to avian strains [4]. The avian virus is more likely to establish infection in swine [15,16]. Evidence for human strains being transmitted to swine was documented when a swine flu H1N2 outbreak in Japan’s pigs was determined to be a result of reassortment between swine H3N2 and human H1N1 [17].

Mathematical models have been used to predict epidemics and develop intervention strategies [18-20]; however, these models have not been developed to include both recombination and cross-species transmission. Here we present a theoretical model that tracks influenza transmission dynamics within and among three species (i.e., birds, pigs, and humans). The model describes how new “super-strains” (i.e., strains that are highly virulent to humans) can arise if pigs act as “mixing vessels” for the recombination of species-specific strains. We use the model to determine the epidemiological outcomes of a reassortant super-strain by varying the factors that influence the strain’s incidence in humans (i.e., transmissibility within humans, pig-to-human transmissibility, and pig-to-human contact). Furthermore, we determine the state(s) (or parameter ranges) of a super-strain that will result in (i) the greatest single epidemic and (ii) the highest cumulative prevalence.

## Methods

**Fig. 1:**
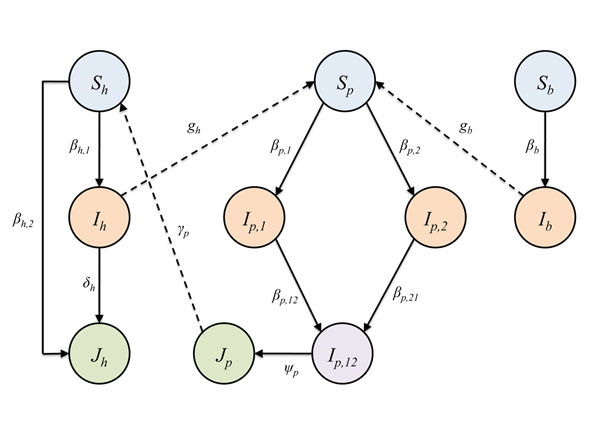
**Schematic diagram illustrating the MSMH model.** Susceptibles are denoted by *S*, infectious classes by *I*, and super-strain infectious classes by *J*. Each species’ classes and parameters for are subscripted by *b* (birds), *h* (humans), and *p* (pigs). All host species have a SIS structure, where recruitment is logistic of the entire population into the susceptible subclass and transfer from infected subclasses by recovery into the susceptible subclass or disease-induced death. The birds have a basic structure, humans have a super-infection structure, and pigs have a coinfection structure with recombination. Solid arrows represent the transfer due to infection within a single host population. Dashed arrows represent the direction of interspecies infectivity from human-to-pig with the endemic human strain, bird-to-pig with the endemic avian strain, and pig-to-human with the super-strain. A description of the variables and parameters is given in Sections 1 and 3 of the Appendix.

To study the emergence of super-strains from interspecies sources, we have constructed a novel multi-strain/multi-host (MSMH) model [21]. This enables the transmission of influenza to be tracked within and between species. We characterize a super-strain in humans as a strain that is highly virulent and capable of directly infecting all humans (i.e., non- and seasonal infected humans) once the strain emerges. Furthermore, when a human with the seasonal strain acquires the super-strain, the super-strain is dominant and there is no coinfection. We use the model to analyze the consequences of a super-strain that forms from a highly virulent (but perhaps non-transmissible) avian strain (e.g., H5N1) and seasonal human strain. A flow diagram for this model is shown in Fig. 1. The avian model (signified by subscript *b*) consists of susceptibles (*S_b_*) and infectives (*I_b_*); the human model (signified by subscript *h*) consists of susceptibles (*S_h_*), seasonal infectives (*I_h_*), and super-strain infectives (*J_h_*); and the swine model (signified by subscript *p*) consists of susceptibles (*S_p_*), seasonal human and avian strain infectives (*I_p,1_* and *I_p,2_*, respectively), coinfected pigs (*I_p,12_*), and infectives that carry a super-strain from recombination during coinfection (*J_p_*). The three host species are coupled as an interacting species system, where the couplings enable avian and human strains to infect pigs (i.e., pigs act as intermediate hosts) and super-strains (developed in pigs) to infect humans. A pig coinfected with both avian and human strains is a “mixing vessel” and can produce a super-strain capable of infecting humans. The ten equations that specify the model, as well as a more detailed description, are given in Section 1 of the Appendix.

We calibrated the MSMH model using data on humans and swine in Southeast Asia. The two human infectious classes are parameterized to represent individuals infected with a seasonal strains (e.g., H1N1 and H3N2) and a transmissible super-strain with disease virulence similar to the H5N1 strain (i.e., 68 percent disease-induced mortality rate) [8]. We chose reasonable parameter values for human influenza transmissibility in Thailand [8,22] and set similar values to sustain prevalent levels of avian and human reassortants in pigs [21]; see Section 3 of the Appendix. Population growth parameters and initial population densities for humans and pigs were determined from census data in Thailand for the twentieth century; see Section 3 of the Appendix. We assume the rate in which a coinfected pig produces a super-strain *ψ_p_* is 0.1. We define the pig-to-human contact transmissibility *γ_p_* as the probability per unit time that a super-strain infected pig will come into contact with and infected human. That is, *γ_p_* is a product of two factors: (i) the rate at which a human will come into contact with a pig and (ii) the rate at which a pig will successfully infect a human with the super-strain. Similarly, the parameters *β_h,1_* and *β_h,2_* are the transmissibility of the seasonal and super- strains, respectively, to susceptible humans (*S_h_*). The transmissibility of the super-strain to seasonal infected humans (or super-transmissibility) is *δ_h_*, which we will assume is equal to *β_h,2_* (i.e., super-strain transmission is the same for susceptible (*S_h_*) and seasonal infectives (*I_h,1_*)). The basic reproductive number *R_0_* for each strain is the average number of secondary infections that one infectious individual can generate in an entirely susceptible population [23]. The *R_0_*'s for endemic strains (i.e., excluding the super-strain in humans) in each species range between 1 and 2.5. We examine *R_0_* values from 0 to 9.5 for a super-strain in humans [18] together with pig-to-human contact transmissibility ranging from 0 to 100 percent, which allows us to consider all epidemiologically relevant cases for past influenza pandemics.

## Results

**Fig. 2:**
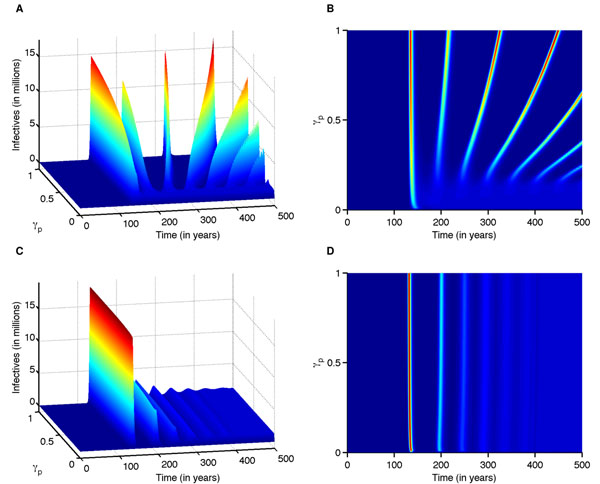
**Time-series figures for the super-infectious humans at varying levels of interaction(A)** The simulation illustrates that for low super-transmissibility (*R_0_*=1.4) and varying levels of interaction (*γ_p_*), the system will exhibit reoccurring outbreaks. **(B)** Bird’s-eye view of the Fig. 2A shows as the level of interaction increases, the outbreaks become larger but further apart. **(C)** The simulation illustrates that for high super-incidence (*R_0_*=2.3) and varying levels of pig-to-human contact transmissibility (*γ_p_*), the system will exhibit an initial large-scale outbreak that is followed by successively damping outbreaks. **(D)** Bird’s-eye view of Fig. 2C shows how varying level of interaction has no effect on the dynamics of the outbreak.

**Fig. 3:**
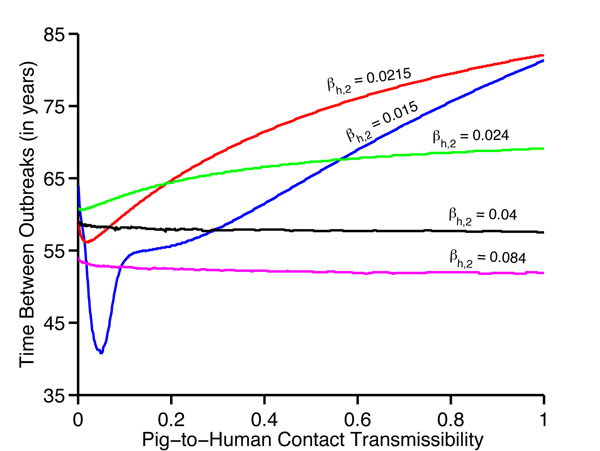
**Time between the first and second outbreaks at varying degrees of pig-to-human interaction** The transmission of the super-strain in humans was set at five specific values. Time frames vary for *β_h,2_*=0.015 from 41 to 81 years (blue), *β_h,2_*=0.0215 from 56 to 82 years (red), *β_h,2_*=0.024 from 61 to 70 years (green), for *β_h,2_*=0.04 from 58 to 59 years (black), for *β_h,2_*=0.084 from 52 to 54 years (magenta). Overall times range from 41 to 82 years.

We simulate the MSMH model over different time periods (i.e., 200-1000 years) to understand both the initial impact and long-term consequences of super-strains. We assume that initially there are neither infected pigs nor super-infected humans. In other words, an initial pre-epidemic period occurs before the super-strain emerges in humans, during which the avian and human strains can cocirculate in pigs for many years or even decades before a super-strain emerges in humans (Figs. 2A-D). The initial outbreak is abrupt once the super-strain emerges in humans and typically has the greatest magnitude. After a super-strain has emerged in humans, there are three possible epidemiological outcomes: periodic outbreaks (Figs. 2A-B for approx. *γ_p_* > 0.15); a super-strain that sustains at low levels without causing a significant outbreak (Figs. 2A-B for approx. *γ_p_* < 0.15); or a strong initial epidemic followed by weaker epidemics (Figs. 2C-D), where the super-strain replaces a previously circulating strain. The outcome is determined by transmissibility of the super-strain and the contact transmissibility from super-infectious pigs to humans. The time frame between the first two outbreaks ranges from 41 to 82 years, depending on the level of transmissibility (Fig. 3).

**Fig. 4:**
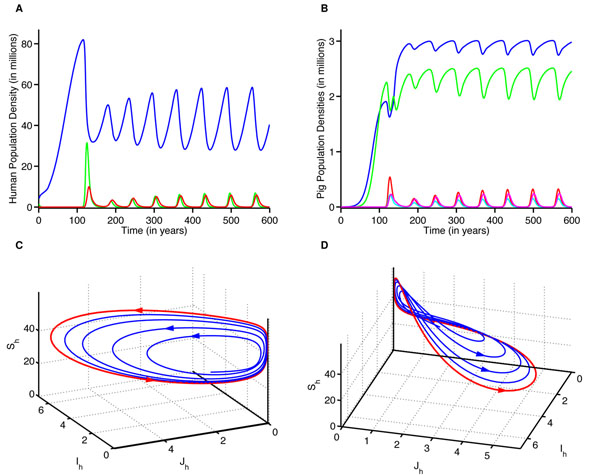
**Simulation of the MSMH model for population densities versus time with high pig-to-human contact transmissibility and low super-strain transmissibility(A)** The simulation for the humans. Colors represent densities over time of the susceptible (blue), primary strain infectious (green), and super-strain infectious (red) classes. **(B)** The simulation for pigs. Colors represent population densities over time of the susceptible (blue), human strain infectious (red), avian strain infectious (green), coinfected (cyan), and super-strain infectious (magenta) classes. **(C)** Phase portrait for the humans. The figure shows human populations (blue) will reach a positive oscillating state (red). **(D)** A different view of the humans’ phase portrait showing the attracting region in 3D space.

When the super-strain is not highly transmissible (i.e., less transmissible than seasonal flu) in humans, the super-strain can cause periodic epidemics in humans (Figs. 2A-B). For *R_0_*= 1.4, periodic outbreaks occur at higher levels of pig-to-human contact transmissibility (approx. γ_p_ > 0.15). In this case, we observe periodic epidemics in all population classes in both humans (Fig. 4A), and pigs (Fig. 4B). In addition, recombinant strains emerge in cycles (cyan: Fig. 4B). The oscillations in the number of coinfected pigs correlate with those of pigs infected with each separate strain (Fig. 4B). Super-infectious pigs attain a maximum number when pigs infected with the human strain are at a peak (cyan: Fig. 4B). Smaller outbreaks are more frequent with lower levels of interaction between pigs and humans, and larger but less frequent outbreaks occur at higher levels of interaction (Fig 2B). The periodicity of each strain will eventually stabilize, and outbreaks of both strains will occur abruptly after interepidemic periods of virtually no disease prevalence (red: Figs. 4C-D. The time between the first two outbreaks ranges from approximately 55 to 81 years and is dependent on the transmissibility of the super-strain and pig-to-human contact transmissibility (Fig. 3: blue). These simulations show the reoccurring nature of pandemics (e.g., the three major outbreaks of the twentieth century: 1918, 1957, and 1968). It is worth noting that the 1918 virus spread between pigs and humans [24]; however, it is unclear whether the virus initially spread from pig-to-human or human-to-pig.

At lower levels of transmission, we found that super-strains were continuously produced in the pig population, but only sustained at a low level (Figs. 2A-B) so they did not pose a threat to the human population. This example correlates to cases when the virus infects a few but is unable to establish itself after the initial outbreak, e.g., the “New Jersey” Incident in the USA in 1976 [25] and perhaps other outbreaks involving a few individuals as described in [4].

When the super-strain is highly transmissible (i.e., approximately as transmissible as 1918 flu, *R_0_*=2.3), the strain will slowly emerge and cause an “epidemic spike” in humans (Figs. 2C-D). In this case, the resulting outbreak dynamics are influenced predominately by the high transmissibility of the super-strain. A series of dampening outbreaks follow the initial spike with periods of virtually no disease prevalence in humans between epidemic outbreaks. The super-strain outcompetes the preexisting endemic strain, causing the endemic strain to become extinct in humans and hence disappear from the pigs, eliminating the reoccurrence of coinfection and recombination. The time frame between the first two outbreaks ranges from approximately 61 to 69 years (Fig. 3: green). These simulations show features present in the pandemics of 1918, 1957, and 1968 in that after the initial pandemic the new super-strain displaces the previously dominant strain [26], continues to circulate, and becomes endemic but with reduced epidemiological impact.

**Fig. 5:**
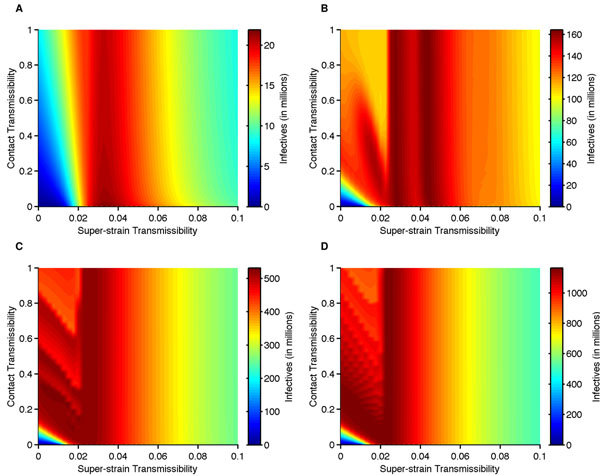
**Contour maps of the pigs-to-human contact transmissibility (*γ_p_*) versus the super-transmissibility in humans (*β_h,2_*)(A)** The figure shows maximum number of super-strain over 200 years. The largest outbreaks occur when 2.3 <*R_0_* < 3.8 (0.24 <*β_h,2_* < 0.4). **(B)** The figure shows the sum of yearly average super-strain cases over 200 years. The two maximal states occur when 2.5 <*R_0_* < 3.0 (0.26 <*β_h,2_* < 0.32) and 4.0 <*R_0_* < 4.5 (0.42 <*β_h,2_* < 0.48). **(C)** The figure shows the sum of yearly average super-strain cases over 500 years. The long-term cumulative incidence of a super-strain is highest when either: 1.9 <*R_0_* < 3.0, or 0 <*R_0_* < 1.9 and a specified range of interaction for the given *R_0_*. **(D)** The figure shows the sum of yearly average super-strain cases over 1000 years. The long-term cumulative incidence of a super-strain is highest when either: 1.9 <*R_0_* < 3.0, or 0 <*R_0_* < 1.9 and a specified range of interaction for the given *R_0_*.

We compare both the epidemic magnitude and cumulative density for varying levels of pig-to-human contact transmissibility and super-strain transmission in humans. By analyzing the largest outbreak for approximately 75 years after the strain has emerged, we determine that a large scale epidemic will occur when 1.9 <*R_0_* < 6.6; however, the greatest epidemic will occur when 2.3 <*R_0_* < 3.8 (Fig. 5A) and not when R_0_ > 3.8. The contact transmissibility does not influence the magnitude of the largest outbreak but is a necessary component for the super-strain to emerge in humans. We determine that a significant outbreak is unlikely for *R_0_* < 1.9 and a moderate outbreak will occur when R_0_ > 6.6. Furthermore, the most significant outbreaks (i.e., 2.3 < R_0_ < 3.8) occur when the super-strain is highly transmissible, and dampened outbreaks follow the large-scale epidemic (Figs. 2C-D). These results for the different values of *R_0_* remain consistent for much longer time frames (e.g., 500 and 1000 years). Our results show that a super-strain that emerges from pigs to humans typically has the greatest potential for human mortalities during its initial outbreak.

A similar analysis of cumulative (averaged annually) infections over different time frames shows higher levels of disease prevalence at lower levels of transmissibility. For 200 years, highest cumulative number occurs when 0 <*R_0_* < 7.6 (Fig. 5B: red-orange), of which there are two maximal states when 2.5 <*R_0_* < 3.0 and 4.0 <*R_0_* < 4.5 (Fig. 4B: dark red). In this case, the cumulative number of infectives can be high even when *R_0_* < 1.9 but depends on the pig-to-human contact transmissibility. For much longer time frames of 500 years (Fig. 5C: dark red) and 1000 years (Fig. 5D: dark red), the maximal prevalence occurs when: 1.9 <*R_0_* < 3.0 for all levels of interaction, and *R_0_* < 1.9 with a sufficient level of pig-to-human contact transmissibility. In fact, long-term prevalence is high even when *R_0_* < 1 (*β_h,2_* < 0.011) and the pig-to-human contact transmission is low (*γ_p_* < 0.4, where the lower bound of *γ_p_* is dependent on the value of *R_0_*). Furthermore, the epidemiological outcome can either be a large-scale outbreak or milder reoccurring outbreaks to sustain maximal prevalence of a super-strain.

## Conclusions

Cross-species interaction and transmission of influenza creates a reservoir of super-strains in pigs; therefore, the continuous introduction of super-strains into humans is almost inevitable. Our modeling is consistent with this notion in that it shows how a super-strain is likely to emerge in humans from an intermediate host, such as pigs. However, we have found that, once a super-strain has emerged, three different outcomes are possible: (i) large scale outbreak, (ii) milder reoccurring outbreaks, and (iii) no outbreak. Examples of each types of outbreak have been well observed in nature. Our results show there may be little to no infectious activity during interepidemic periods; hence, epidemic forecasting may be difficult until the early stages of an outbreak. This is particularly important to ensure vaccine development in that detection may not be possible until an outbreak is significant. The more recent 2009 H1N1 strain was a reassortant of avian, human, and swine strains, and its gene segments have been circulating undetected for an extended period [7]. Our results also show that the greatest outbreak occurs when 2.3 <*R_0_* < 3.8, which may be a consequence of strain competition and interspecies infection. That is, pig-to-human infection provides a competitive advantage to the super-strain over the seasonal. Higher *R_0_* values for the super-strain may result in competitive exclusion of the seasonal strain, thus suppressing seasonal infections and limiting the opportunity for external infections.

We have demonstrated how genetic recombination and species interactions significantly affect the emergence and dynamics of influenza strains. The notion of reoccurring epidemics is particularly important in that it is specifically driven by species interaction and may explain the disappearance and re-emergence of a subtype many years after an epidemic. We recommend, that in order to obtain further insights into the emergence of influenza super-strains, strain subtypes should be frequently monitored in the major host species (particularly, birds, humans, and pigs). In addition, we recommend comparing various reassortants to identify strains that are more likely to recombine, particularly those that are infectious to humans.

A reassortant of the avian strain such as the H5N1 that can spread from human-to-human poses a great threat, even when transmissibility is low (as shown in our simulation). Although the chance of such an event seems rare, situations like one described in the report [13] on Java’s pigs suggest that not only is a super-strain able to emerge but that such a strain is likely to emerge. Our results suggest more careful surveillance of influenza in pigs with a specific focus on detecting rare but potentially dangerous recombination events. Unless a global monitoring system is put in place, we will be unable to recognize lurking novel strains that are capable of the next pandemic. Most importantly, our modeling shows the necessity of a global monitoring system to track strain subtypes within different host species to adequately forecast the next pandemic.

## Abbreviations

FAO, Food and Agriculture Organization of the United Nations; MSMH, multi-strain/multi-host; SIS, susceptible-infective-susceptible, WHO, World Health Organization.

## Competing interests

The authors declare that they have no competing interests.

## Authors’ contributions

BJC, CC, and SR developed the concept and study design, analyzed and interpreted the data and drafted the manuscript. BJC conducted mathematical analyses and simulations.

## Appendix

### Multi-strain/multi-host model

We use the multi-strain/multi-host (MSMH) model to simulate the spread of influenza in bird, pigs, and humans. Each species has a set of susceptible-infectious-susceptible (SIS) type differential equations that govern the spread of certain strains in that population. Moreover, some host species can infect other species with specific strains, but this ability is generally not symmetric. For example, birds can infect pigs with an avian strain, but the pigs cannot pass the avian strain back into the avian population. All infections follow a “mass action” structure, and recruitment is into the susceptible subclass, so the birth term is logistic and based on the entire population. The three host species are coupled by external inputs of avian and human strains from the respective hosts to the pigs and a super-strain external input from the pigs to the humans.

The subscripts *b*, *p*, and *h* denote birds, pigs, and humans, respectively. *S_i_(t)* is the density of susceptibles for species *i* at time *t*; *I_i_,_j_(t)* is the density of *j*th infectious individuals for species *i* at time *t*; and *J_i_(t)* is the density of super-infectious individuals for species *i* at time *t*. *β* is the per capita incidence rate, *α* is the recovery rate, *v* is the disease-induced mortality rate (virulence). *r* is the intrinsic growth, *K* is the carrying capacity, and *N* is the total population. More specifically, *N_b_=S_b_+I_b_*, *N_p_=S_p_+I_p,1_+I_p,2_+I_p,12_+J_p_*, and *N_h_=S_h_+I_h_+J_h_*. *δ_h_* is the super-transmission rate in which super-infectious humans *J_h_* infect individuals in the group *I_h_*. *Ψ_p_* is the rate at which co-infected pigs become super-infected. *g_b_* and *g_h_* are the transmission rates in which birds and humans infect pigs, respectively. *γ_p_* the transmission rate super-infectious pigs infect humans.

### Basic reproductive numbers

In this section, we calculate the basic reproductive numbers *R_0_* for the MSMH model. In general, *R_0_* for an independent strain (i.e., when the MSMH model is reduced to a species’ susceptibles and specified infectious individual) is

For example, the basic reproductive number for the independent super-strain in humans is

We calculate *R_0_* for the MSMH model by using the methods for compartmental models provided in [27]. We calculate about the disease-free equilibrium point *(K_b_,0,K_p_,0,0,0,0,K_h_,0,0)*. The next-generation matrix (*FV^-1^*) is

The basic reproductive number (i.e., the spectral radius of *FV^-1^*) for the MSMH model is

which is the maximum basic reproductive number of the strains in the three host populations.

### Parameter values

To calibrate the human parameters, we set the parameter values for the two strains in humans to reflect a seasonal strain and a transmissible avian strain with the virulence of H5N1. We use data from Thailand because of the surveillance of strains and the prevalence of the avian strain H5N1. To determine the logistic growth parameters in humans, we use data for the total population of Thailand [28,29]. We best-fit the data to the solution of the logistic equation given by

using the method of least squares (Fig. S1). The parameters were determined using iterative methods: *K_h_*=87.3, *S_h_(0)*=3.983, and *r_h_*=0.038. In a study by the National Institute of Health in Thailand [22], case specimens of influenza were taken from patients in 2004 and 2005. It was determined that the numbers of cases of influenza-like illness for 2004 and 2005 were 21,176 and 21,351 per 100,000 people, respectively. The study also sampled specimens for influenza type and subtype. We determined that the incidence *β_h,1_* for the endemic strain of influenza is 0.0253 and 0.0273 for the years 2004 and 2005, respectively. The incidence *β_h,2_* of the avian strain H5N1 for 2004 was determined to be 0.00084. A summary report on influenza in Asian countries from 1999 cites the annual mortality due to pneumonia as 176 per 100,000 people [30], so we let the disease-induced mortality rate be *v_h,1_*=0.00176 and the recovery rate *α_h,1_*=0.99824. The World Health Organization (WHO) reported on June 2008 that the number of confirmed cases due to H5N1 in Thailand was 25 and the number of deaths was 17 [8], so let the disease-induced mortality of the super-strain to be *v_h,2_*=0.68 and recovery rate *α_h,2_*=0.32.

To calibrate the pig parameters, we used data from the Food and Agriculture Organization of the United Nations (FAO) on pig populations [31,32] and fit the data using the same methodology as in the humans (see above). The parameters for the logistic growth term were determined to be *K_p_*=9.16, *S_p_(0)*=0.002, and *r_p_*=0.093. Due to the limited data on influenza in pigs, we assumed reasonable values (i.e., similar to the values for humans) for the remaining parameters. We assumed the birds have the same parameters as humans; that is, the single strain will have the same values as the endemics strain in humans.
